# Adventures in the Enormous: A 1.8 Million Clone BAC Library for the 21.7 Gb Genome of Loblolly Pine

**DOI:** 10.1371/journal.pone.0016214

**Published:** 2011-01-21

**Authors:** Zenaida V. Magbanua, Seval Ozkan, Benjamin D. Bartlett, Philippe Chouvarine, Christopher A. Saski, Aaron Liston, Richard C. Cronn, C. Dana Nelson, Daniel G. Peterson

**Affiliations:** 1 Mississippi Genome Exploration Laboratory and Department of Plant and Soil Sciences, Mississippi State University, Mississippi State, Mississippi, United States of America; 2 Life Sciences and Biotechnology Institute and Institute for Digital Biology, Mississippi State University, Mississippi State, Mississippi, United States of America; 3 Clemson University Genomics Institute, Clemson University, Clemson, South Carolina, United States of America; 4 Department of Botany and Plant Pathology, Oregon State University, Corvallis, Oregon, United States of America; 5 Pacific Northwest Research Station, United States Forest Service, Corvallis, Oregon, United States of America; 6 Southern Institute of Forest Genetics, United States Forest Service, Saucier, Mississippi, United States of America; King's College London, United Kingdom

## Abstract

Loblolly pine (LP; *Pinus taeda* L.) is the most economically important tree in the U.S. and a cornerstone species in southeastern forests. However, genomics research on LP and other conifers has lagged behind studies on flowering plants due, in part, to the large size of conifer genomes. As a means to accelerate conifer genome research, we constructed a BAC library for the LP genotype 7-56. The LP BAC library consists of 1,824,768 individually-archived clones making it the largest single BAC library constructed to date, has a mean insert size of 96 kb, and affords 7.6X coverage of the 21.7 Gb LP genome. To demonstrate the efficacy of the library in gene isolation, we screened macroarrays with overgos designed from a pine EST anchored on LP chromosome 10. A positive BAC was sequenced and found to contain the expected full-length target gene, several gene-like regions, and both known and novel repeats. Macroarray analysis using the retrotransposon IFG-7 (the most abundant repeat in the sequenced BAC) as a probe indicates that IFG-7 is found in roughly 210,557 copies and constitutes about 5.8% or 1.26 Gb of LP nuclear DNA; this DNA quantity is eight times the *Arabidopsis* genome. In addition to its use in genome characterization and gene isolation as demonstrated herein, the BAC library should hasten whole genome sequencing of LP via next-generation sequencing strategies/technologies and facilitate improvement of trees through molecular breeding and genetic engineering. The library and associated products are distributed by the Clemson University Genomics Institute (www.genome.clemson.edu).

## Introduction

Loblolly pine (LP; *Pinus taeda* L.) is an organism of tremendous economic and ecological importance and a key representative of the conifers, an ancient lineage of plants that dominates many of the world's temperate and boreal ecosystems [1]. LP's fast growth, amenability to intensive silviculture, and high-quality lumber/pulp have made it the cornerstone of the U.S. forest products industry and the most commonly planted tree species in America – approximately 75% of all seedlings planted each year are LPs [2]. Its ability to efficiently convert CO_2_ into biomass and its widespread use as a plantation tree have also made LP a cost-effective feedstock for lignocellulosic ethanol production [3] and a promising tool in efforts to curb greenhouse gas levels *via* carbon sequestration [4].

Despite the importance of LP and other conifers, genomic sequence information for this taxon is extremely limited. Like other conifers, LP has a relatively huge genome – its 1C DNA content is reported at 21.7 Gb [5]. Its long generation time, approximately eight years to sexual maturity, also poses an obstacle to tree improvement through traditional breeding techniques. Though molecular resources such as genetic maps [6]–[8], a FISH-based karyotype [9], EST sequences [10]–[13], and QTL maps are available [6]–[8] for LP, efficient tree improvement will ultimately require integration of EST, sequence polymorphism, gene expression, and genetic data with actual genomic sequence including non-coding regulatory regions missed by EST approaches.

To accelerate pine genomics, we constructed and initiated characterization of a bacterial artificial chromosome (BAC) library for the LP tree “7-56,” a valuable and widely used parent selection in various loblolly pine breeding programs [14]. The utility of the library for gene isolation and genome characterization was verified by macroarray analysis and DNA sequencing. The 7-56 BAC library is a high quality resource that will expedite research on pine and conifers in general.

## Results and Discussion

### Library construction and characterization

**Figure 1:**
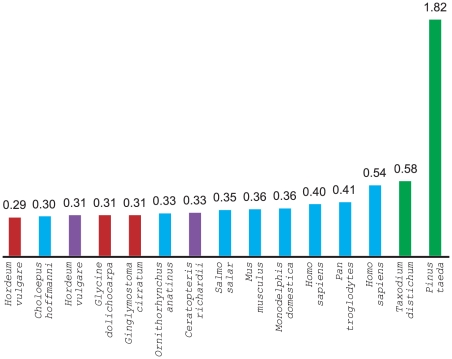
Clone numbers (in millions of clones) for the largest BAC libraries. The *Pinus taeda* BAC library is three times larger than the second largest library. Bar colors represent the center/institute at which the library was made – red represents the Arizona Genomics Institute (AGI; www.genome.arizona.edu), blue the Children's Hospital of Oakland (CHORI; http://bacpac.chori.org), purple the Clemson University Genomics Institute (CUGI; www.genome.clemson.edu), and green the Mississippi Genome Exploration Laboratory (MGEL; www.mgel.msstate.edu).

The completed library consists of 1,824,768 clones archived in 4752 384-well microtiter plates – to our knowledge this is the single largest BAC library ever made (see Figure 1). Two sets of replicate libraries were prepared and stored in separate −80°C freezer banks at the Mississippi Genome Exploration Laboratory (MGEL; www.mgel.msstate.edu) while the original was sent to the Clemson University Genomics Institute (CUGI; www.genome.clemson.edu) for distribution and remote storage. For distribution, the library has been gridded onto macroarrays using a 5×5 format in which 27,648 clones are double-spotted on each 22 cm^2^ membrane. A complete set of macroarrays consists of 66 filters. However, screening of the library at MGEL was performed using macroarrays with a 4×4 gridding pattern (i.e., 18,432 double-spotted clones on each 22 cm^2^ macroarray).

**Figure 2:**
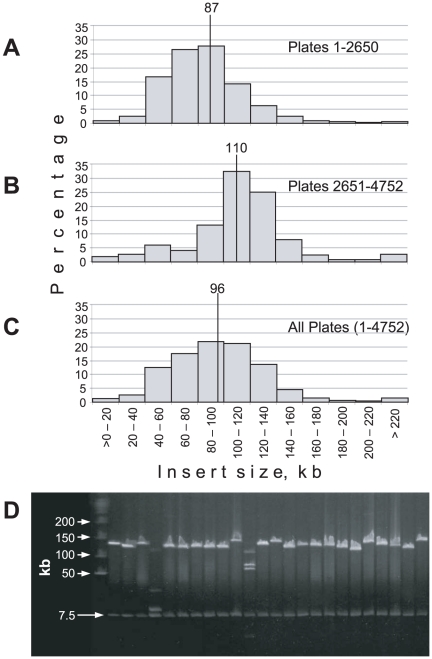
Inserts of LP 7-56 BAC clones. Insert size distribution of clones from (A) plates 1-2650, (B) plates 2651-4752, and (C) the library as a whole. (D) A typical agarose pulsed-field gel showing *Not*I digests of clones from the latter half of the library. The New England Biolabs PFGE Lambda Ladder is in the lane at the far left. A 7.5 kb vector band is visible at the bottom of each sample lane.

Unlike other plant and animal species for which we typically obtain clones with mean insert sizes in excess of 100 kb with only modest optimization, application of standard BAC library construction protocols resulted in LP clones with mean insert sizes <75 kb. We used a variety of techniques to increase insert size including varying tissue sources, the enzymes for partial restriction digestion, the cloning vectors, the vector to insert ratio used in ligation, and the ligase concentration. Many of these steps led to minor increases in mean insert size, but ultimately were not sufficient to provide mean insert lengths ≥100 kb. The breakthrough that permitted realization of the >100 kb mean insert size goal came with the discovery and adoption of the “pre-electrophoresis” procedure of Osoegawa et al. [15]. In pre-electrophoresis, agarose plugs containing DNA are placed in a dialysis tube, the tube is positioned in the center of a pulsed-field gel electrophoresis (PFGE) chamber, and the tube is exposed to a voltage that permits charged low molecular weight molecules to migrate out of the plugs. Pre-electrophoresis has been used to remove residual proteins from DNA plugs to help prevent inhibition of downstream processes such as ligation [15]. In our experience, it appears to elute much of the low molecular weight DNA from the plugs which, in turn, appears to enhance restriction enzyme digestion and fragment separation via PFGE. Clones from the first 2650 384-well plates had an average insert size of 87 kb based on *Not*I digestion and PFGE analysis. However, with the addition of the pre-electrophoresis step, mean insert size was increased to 110 kb for plates 2651-4752. The insert size distributions of clones are shown in Figures 2A–2C. The average insert size of the entire library is estimated at 96 kb. A typical gel containing *Not*I-digested clones from the latter half of the library is shown in Figure 2D. Probing of a Southern blot of *Not*I-digested clones with LP 7-56 genomic DNA was used to confirm that library inserts were indeed derived from pine (data not shown).

Of note, LP DNA appears to contain relatively few *Not*I sites as more than 80% of the *Not*I-digested clones examined yield a single insert band (Figure 2D). In this regard, the LP clone *Not*I digestion pattern is similar to those observed for BAC libraries from dicotyledonous plants; monocots typically possess much higher densities of *Not*I sites [16]. Based on examination of 95 gels (7626 clones), we estimate that the LP genome contains an average of one *Not*I site per 1000 kb of sequence.

As is standard in evaluating plant BAC libraries, we estimated the fraction of clones that lack inserts (i.e., false positives) and the fraction that contain chloroplast DNA. PFGE results indicate that roughly 5.7% of clones appear to be false positives. Hybridization of a 4×4 macroarray with pine chloroplast DNA probes revealed that about 0.6% of the LP clones contain chloroplast DNA, a mid-low level of chloroplast contamination compared to other plant BAC libraries (range: 0.02–2.78%; see [17]–[28]). While macroarray screening with mitochondrial DNA was not performed, automated analysis of Sanger and 454 sequence reads [29] prepared from LP 7-56 nuclear DNA using our nuclear isolation protocol revealed that mitochondrial DNA contamination is 10 to 100 times less frequent than contamination from chloroplast DNA (unpublished data).

Assuming that 0.057 of clones are false positives and 0.006 of clones contain chloroplast DNA, the number of clones containing pine nuclear DNA is approximately [i.e., (1-(0.057+0.006))*1,824,768 = ] 1,709,808. Since the LP genome is 21.7 Gb [5], a library containing 1,709,808 pine nuclear DNA-containing clones with 96 kb inserts affords coverage of roughly 7.6 genome equivalents [i.e., (1,709,808 clones • 96,000 bp) ÷ 21.7×10^9^ bp  = 7.6]. A 7.6X library affords a 99.93% probability that any locus of interest will be found in the library at least once [30].

### Identification, sequencing, and annotation of a gene-containing BAC

**Figure 3:**
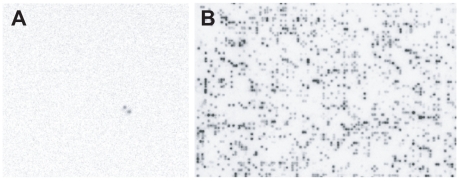
Screening LP 7-56 BAC macroarrays. (A) Use of the BAC library in gene isolation. The autoradiogram shows hybridization of an overgo probe linked to the LEA gene on LP chromosome 10 to a double-spotted BAC clone potentially containing a full length LEA gene. The positive BAC clone, PT_7Ba_00066 J18, was sequenced and indeed found to contain an intact LEA gene. (B) Hybridization of a 4×4 membrane with the IFG-7 retroelement. Note that IFG-7 is found in many, but not all BAC clones. Also note that some clones appear to contain higher densities of the retroelement.

A major use of BAC libraries is in the isolation of intact genes including non-coding regions missed by cDNA/EST sequencing approaches [16], [31]. To demonstrate the utility of the library for this task, we selected an EST (GenBank AA739884) that has been mapped to LP chromosome 10 [10] and displays significant homology (S' = 392) with a *Picea glauca* late embryogenesis abundant (LEA) protein. Overgos designed from the marker were used to screen the first two 4×4 macroarrays of the library (Figure 3a), and PCR was used to check for the presence of the marker in clones exhibiting probe hybridization. A positive clone, PT_7Ba_00066 J18, which has an insert size of 86.5 kb, was sheared, bar coded, and added to a solution containing numerous differentially bar coded chloroplast genomes. From relatively low-coverage (20X) Illumina sequencing, the BAC was assembled into 158 contigs (not including vector contigs) with a combined length of 85,504 bp, i.e., roughly 98.8% of the estimated insert size. The sequences that resulted from this BAC were deposited to GenBank (Accession Number HQ141589). A 4048 bp contig in the BAC contains the target full-length LEA gene (see below for more information).

**Figure 4:**
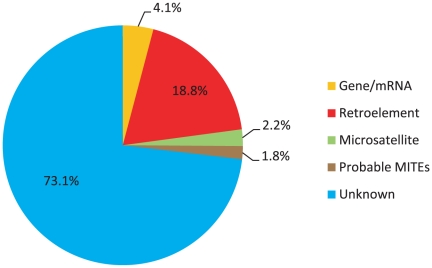
BLAST-based analysis of the LP 7-56 clone PT_7Ba_00066 J18 sequence.

Figure 4 summarizes the results of an initial sequence analysis of the BAC. The majority of the BAC sequence exhibits no recognizable homology to any annotated plant sequences in GenBank. With regard to retroelements, our results indicate that long-terminal repeat (LTR) retrotransposons account for at least 18.8% of the total BAC length with the majority of such sequences showing their most significant (S' >50) homology to the previously described Gypsy subfamily LTR elements IFG-7 [32], PpRT1, [33], and/or Corky (GenBank Accession No. EU862277.1). No recognizable Copia subfamily LTR element was found in the BAC, and indeed our analysis of this BAC coupled with characterization of random sequences we have generated from pine via 454 and capillary sequencing indicates that the LP genome contains far more LTR Gypsy elements than LTR Copia elements (unpublished data). DNA transposons were not identified by homology (BLAST; [34]) searches. However, using the program FINDMITE [35] we identified 122 potential miniature inverted-repeat transposable elements (MITEs) in the BAC. MITEs are non-autonomous DNA transposons characterized by terminal inverted repeats, target site duplications, and no coding sequence [36]. Three of the putative MITEs appear to be portions of retroelements. The other putative MITEs are currently being further investigated, though it is probable that most of these sequences do not actually represent true MITE families. However, four instances were found where a MITE recognized by FINDMITE exhibited >80% sequence identity to another region in the BAC not recognized by the FINDMITE program. These instances may represent four different MITE families where duplicated copies have undergone moderate divergence, and indeed these sequences are priorities in our MITE investigations.

Putative genes, i.e., sequences exhibiting significant alignment (S' ≥50) to known genes and/or cDNA sequences, constitute approximately 4.1% of the BAC sequence (including the putative LEA gene – see below). However, only the LEA gene appears to have a complete coding sequence.

### Annotation of the LEA gene

**Figure 5:**
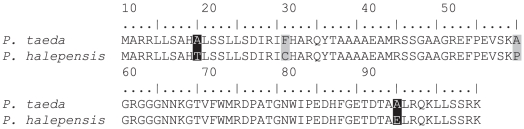
Alignment of the predicted amino acid sequences of the *Pinus taeda* 7-56 LEA gene (top) and the *Pinus halepensis* 15r LEA mRNA (bottom; GenBank Accession No. AY705798.1) suggest that the proteins differ at four amino acids (highlighted residues). Interspecific single nucleotide differences highlighted in light gray do not result in a change in overall polarity and/or charge. However, differences highlighted in black result in a polarity and/or charge difference (e.g., A =  nonpolar, neutral; T  =  polar, neutral; E  =  polar, negative).

The targeted LEA gene sequenced in this study (i.e., LBAC) was found within a 4026 bp contig. A BLAST (blastn) comparison of the contig with the NCBI non-redundant (nr) database produced a top hit (S' = 675) to the complete coding sequence of a *Pinus halepensis* LEA mRNA (GenBank Accession No. AY705798.1). Examination of the aligned portions of LBAC with the *P*. *halepensis* LEA mRNA suggests that LBAC is composed of two exons and one intron (see Figure S1). The coding sequences from the two species are the same length but contain six interspecific single nucleotide differences (Figure S1) which are predicted to result in four amino acid differences between the predicted *P*. *taeda* and *P*. *halepensis* proteins (Figure 5).

BLASTX analysis of coding sequence of the LP LEA gene indicates that a 50 amino acid region (positions 43 through 92) exhibits significant homology (S' = 68) to pfam03242 [37], the LEA 3 family of proteins (Figure S2). The LEA proteins, including the LEA 3 family, have been implicated in response to water stress, though the exact function of these proteins is not clear [38]–[41].

### Comparison of the LBAC gene and ESTs

The LBAC gene was also aligned with *Pinus taeda* sequences in the NCBI non-human, non-mouse EST database (est_others). The top 250 blastn hits (S' = 719 to 1700) produced via blastn comparison fall into two structurally distinct groups.

#### Variant 1

Exactly 239 of the 250 top hits have exon sequences that are 100% identical to the LBAC exons. Of these, six (i.e., 2.5%) contain a putative intron in their sequences; each of these introns is identical to the LBAC intron. Consequently, it is probable that the 239 variant 1 ESTs are products of the LBAC locus. Moreover, the presence of variant 1 transcripts with and without an intron indicates that there is a certain level of alternative and/or inefficient splicing of LBAC/variant 1 transcripts. A consensus sequence including the intron (deemed Consensus Sequence, Variant 1 or CSV1; see Figure S1) was generated for the 239 variant 1 ESTs. CSV1 is identical to the LBAC sequence.

#### Variant 2

Eleven of the EST sequences exhibited consistent sequence differences from LBAC and CSV1. As shown in Figure S1, the consensus sequence for these variant 2 sequences (i.e., Consensus Sequence, Variant 2 or CSV2) possesses 20 single nucleotide differences when compared to CSV1. Moreover, all of the variant 2 sequences possess the region marked as an intron in CSV1/LBAC. Of note, this region of variant 2 ESTs contains a 12 nt deletion which may account for the apparent 100% retention of the intron in variant 2 transcripts.

While we speculate that the variant 1 sequences are products of the LBAC gene, it is unclear whether variant 1 and variant 2 transcripts represent products of different alleles of the same gene or products of paralogous genes. Of note, the sequenced *Pinus halepensis* LEA mRNA (GenBank Accession No. AY705798.1) lacks the putative intron (as with most variant 1 sequences) but shares a 13 nt insertion immediately after the stop codon with variant 2 ESTs (Figure S1).

### Use of the LP BAC library to characterize the IFG-7 retrotransposon

A BAC library is an excellent resource for the initial characterization of a genome, especially in cases when sequence information is limited [42]. All plant genomes studied thus far contain numerous transposable elements, and the proportion of these elements in genomes tends to increase with genome size [43]. To begin genome characterization of LP, we chose to look at the distribution of IFG-7 [32], the most abundant retroelement in the BAC we sequenced (i.e., PT_7Ba_00066 J18). A 568 bp pine sequence exhibiting 99% sequence identity to the *Pinus radiata* IFG-7 retrotransposon was used to probe a 4×4 LP macroarray. The differences in hybridization intensities between positive clones suggest that higher intensity clones likely harbor multiple copies of the retrotransposon (Figure 3B). Using the densitometry method of Peterson et al. [42] with minor modification (see Materials and Methods), we calculated that there are approximately 210,557 copies of IFG-7 in the LP genome which collectively account for about 5.8% (i.e., 21.7 Gb • 0.058 = 1.26 Gb) of pine nuclear DNA. This amount of DNA is roughly equivalent to about eight *Arabidopsis thaliana* (1C  = 157 Mb; [44]) genomes.

An initial glance at IFG-7 hybridization to macroarrays suggests that the element is found in clusters, i.e., it is not distributed randomly throughout the genome (Figure 3B). To test this hypothesis, we used the probabilistic “urn model” method applied in Shan et al [45] and described in detail in Holst [46]. Using our estimate of the number of copies of IFG-7 in the LP genome (i.e., 210,557), and an average insert size of 96 kb, each macroarray, after adjustment for false positives and chloroplast-containing clones, represents about 0.076X coverage of the LP genome [i.e., (18,432 clones/macroarray • 0.937 nuclear DNA-containing clones • 96 kb)/21.7 Gb  = 0.076] and should contain 16,002 copies of IFG-7 (i.e., 210,557 copies • 0.076 = 16,002). If the distribution of IFG-7 were indeed random, we would expect that the distribution of clones lacking IFG-7 elements (i.e., lacking hybridization signal) would approximate normality; in such cases, the mean number of clones expected to lack an IFG-7 element and the theoretical standard deviation (*SD*) can be estimated using Theorem 2 of Holst [46]. Specifically,
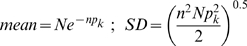
where *N* is the number of nuclear DNA-containing clones on the macroarray (17,271), *n* is the expected number of clones showing IFG-7 hybridization (i.e., 16,002), and *p_k_* is the probability of any copy of the element “falling” into a clone (i.e., 1/17,271). Plugging the values into the equations results in a mean of 6,838 clones per macroarray with a standard deviation of 86. However, our macroarray analysis shows that 13,926 of the 17,271 clones do not exhibit IFG-7 hybridization. Hence, the observed number of clones lacking IFG-7 hybridization is 161 times (13926/86 = 161) the expected standard deviation for a normal distribution, strongly reinforcing our hypothesis that the distribution of IFG-7 is not random. Of note, non-random distributions of transposable elements has been reported for many plant species [47]–[50].

### Utilization of the BAC library in genome sequencing of LP

The U.S. Department of Agriculture recently announced plans to fund draft sequencing of the LP genome. While it is not publicly known how this decision was reached, it is likely that the success of former and current investments by the National Science Foundation, Department of Energy, and USDA in LP genome research, including NSF funding of production of the 7-56 BAC library, created the scientific framework on which such an effort could be justified. If the LP 7-56 BAC library is used in sequencing the LP genome, it is unlikely that traditional BAC-based physical mapping approaches (including BAC end sequencing and BAC fingerprinting) will be employed to a large extent as the LP genome is simply too big for a clone-by-clone, physical map-based sequencing approach to be cost effective. However, the organized nature of a BAC library (specifically the storage of individual clones in indexed plates/wells) affords a mechanism that can be utilized in simplifying sequence assembly. In short, one can sequence pools of BACs *in lieu* of (or preferably, in addition to) random genomic DNA. Each pool contains a specific number of BACs (e.g., 1000) and hence represents a fairly small portion of the genome. The probability of two homologous or paralogous loci being represented within a pool is small, thus limiting assembly problems associated with diploidy/polyploidy and large gene families, respectively. Though a repeat sequence may be found millions of times within a genome, its representation in a BAC pool is likewise greatly reduced as are its effects on assembly of sequences in that pool. Of particular importance, the clones in each pool are archived allowing the pool to be reconstructed if necessary. Moreover, one can further refine the assembly process by using multiplexing strategies to produce partially overlapping BAC pools and/or bar coding individual BACs or BAC subpools (e.g., [51], [52]).

### Conclusion

To accelerate genomics research in pine, we constructed a pine *Hind*III BAC library that affords roughly 8.1X coverage of the LP genome. This resource should allow isolation and sequencing of most pine loci and represents a means of facilitating physical mapping, gene isolation, and genome sequencing. It is anticipated that the BAC library will be a key resource utilized in sequencing the loblolly pine genome.

## Materials and Methods

### Plant material

Loblolly pine genotype 7-56 (origin: Williamsburg County, South Carolina, original tree is deceased) needles were provided by International Paper from a single ramet growing at their Southlands Forest near Bainbridge, Georgia. Prior to selecting this ramet, six short simple repeat (SSR) marker loci – specifically PtTX2123, PtTX4058, PtTX4093, PtTX4181, PtTX3013 and PtTX3052 [53] – and the CAD-null marker [54] were used to genotype ten 7-56 and two non-7-56 ramets in a double blind experiment. Protocols for SSR genotyping are given in Gonzalez-Martinez et al. [55]. All 7-56 ramets, including the one selected as a tissue donor, were found to have the same multi-locus SSR genotype, and this genotype differed from the negative controls. In addition, these SSR genotype data matched our data from previous independent sample collections of 7-56 indicating that these samples were indeed genotype 7-56. Upon harvest needles were wrapped in moist paper towels, placed in large sealable plastic bags, and shipped on ice via overnight courier. Bags of needles were stored at 4°C until use.

### Library construction and storage

Construction of the library was performed according to Peterson et al. [16] with the following modifications:

#### (1) Pre-electrophoresis

Prior to size selections, agarose plugs containing LP genomic DNA were suspended in 0.5X TBE buffer and sealed inside a Spectra/Por MWCO 12-14,000 dialysis tube (Spectrum Laboratories) that was placed in the center of the hexagonal electrode array in a Bio-Rad DRIII CHEF pulse-field gel electrophoresis (PFGE) apparatus. The PFGE unit was run for 8 hours with pulse ramping of 1 to 4 sec, an included angle of 120°, and a voltage gradient of 6 V/cm in 0.5X TBE buffer at 14°C.

#### (2) Size selections

Gold Agarose (Seakem) in 0.25X TBE was used in all size selection steps, along with 0.25X TBE as running buffer. After the first selection step as described in Peterson et al. [16], the gel was allowed to run for an addition 9 h using a 3 s start switch time and a 5 s end switch time. This additional electrophoresis period increases the resolution of separation. Agarose containing DNA molecules between 120 to 220 kb was excised and loaded into a second gel as described [16] except the total run time was 14 rather than 18 h. At the end of the run, only agarose containing DNA in the range of 120 to 220 kb was excised for electroelution.

#### (3) Electroelution

The DNA elution procedure of Peterson et al. [16] was followed except that the process was performed for 2 h, and buffer in the upper chamber of the instrument was replaced every 30 min.

#### (4) Ligation and transformation

The eluted DNA was quantified with a NanoDrop ND-1000 (Thermo Fisher Scientific) spectrophotometer. The ligation and transformation steps were carried out as illustrated in Peterson et al. [16] except that the vector used was *Hind*III-ready pIndigoBAC5 (Epicentre Technologies). Ligation was carried out in a reaction containing 30 ng dephosphorylated vector DNA, 600 ng size-selected insert DNA, 15 µL of 10X ligase buffer, 2 µL of 2,000 units/µL T4 DNA ligase (New England Biolabs), and deionized water to produce a solution with a final volume of 150 µL.

Transformations, picking of colonies, plate replication, and storage of plates at −80°C were performed as previously described [16]. All microtiter plates containing clones were affixed with labels containing alphanumeric and bar code identifiers. The library was given the name PT_7Ba in accordance with MGEL and CUGI naming conventions (see http://www.mgel.msstate.edu/dna_libs.htm).

### Sampling and Analysis of Clones

The average molecular weight of the inserts and the percentage of vectors without inserts were estimated based on sampling of 82 clones from every 50^th^ plate. In brief, for each plate a manual 96-pin plate replicator (V & P Scientific) was used to transfer bacteria from 96 of the wells (offset A) into media in two AutoGen 96-well plates. The wells in the AutoGen plates were filled with 1.2 mL Terrific Broth (Difco) supplemented with 30 µg/mL of chloramphenicol. The plates were incubated at 37°C for 18–24 hours with shaking at 250 rpm. Bacterial cells from duplicate plates were pooled and the BACs were isolated from the cells using an AutoGen Prep 960 (AutoGen) robot. After air drying, recovered DNA pellets each were dissolved in 15 µL of a solution of 1.5 µL of 10 units/µL *Not*I (New England Biolabs), 0.1 µL of 10 mg/mL BSA, 1.5 µL of 10X Buffer 3 (New England Biolabs; 500 mM Tris-HCl, 1000 mM NaCl, 100 mM MgCl_2_ 10 mM dithiothreitol), and 12.9 µL double-distilled water). Digestion was allowed to proceed at 37°C for 5–14 hours. The digested BACs were run on a CHEF gel as previously described [16]. The New England Biolabs Lambda PFGE ladder was used as a standard when estimating the sizes of inserts.

### Gridding and hybridization of high density filters

Macroarrays were prepared using a Genetix QPixII robot. After the clones were spotted onto membranes, they were placed on LB agar trays (clone side up) and allowed to grow overnight at 37°C. Membranes were fixed by incubation in Solution 1 (0.5 N NaOH, 1.5 M NaCl) and Solution 2 (1.5 M NaCl, 0.5M Tris Cl) for 7 min each. The membranes were allowed to dry for 1 h, washed in 0.4 N NaOH for 20 min, and washed for 7 min in aqueous 750 mM NaCl, 50 mM NaH_2_PO_4_, and 50 mM Na_2_EDTA. The membranes were pre-hybridized for at least 3 h, or overnight if they had not been hybridized before, in hybridization buffer (0.25 M Na_2_HPO_4_, pH 7.2; 7% w/v SDS; 1 mM EDTA; and 1% w/v BSA) in a hybridization oven (SciGene, Model 400) using a rotation setting of 4. The membranes were separated by nylon mesh sheets (Fisher Scientific, Pittsburgh, PA) and rolled to fit into hybridization bottles. A maximum of five membranes were placed in each bottle, along with 50 mL of temperature-equilibrated hybridization buffer. Hybridization was carried out at 55°C for overgos and 65°C for longer probes (see below for probe labeling and concentrations used in hybridization experiments).

To identify a BAC clone containing the LEA gene, two macroarrays representing the first 96 microtiter plates of the library were screened with overgo probes designed from an EST marker found on pine chromosome 10 [10]; GenBank Accession No. AA739884). The design and preparation of overgo sequences is described in Table S1. The program *MacroArray Reader*, developed at MGEL (manuscript in preparation), was used to identify the locations of positive clones on the high density membranes. PCR was used to verify the presence of the LEA gene in the PT_7Ba_0006 J18 BAC using EST-specific primers (sequences provided in Table S1).

Pine genomic DNA and chloroplast clones were labeled using the Megaprime DNA Labeling System (GE Healthcare). The genomic DNA was digested with *Hind*III for 2 h, precipitated with ethanol, and dissolved in double-distilled water prior to labeling. The chloroplast probes were obtained from clones, in our possession (unpublished data) that align with nucleotides 27939-28367, 60489-61592, 79999-81133, and 117813-118274 of the 119,707 bp *Pinus thunbergii* chloroplast genome (GenBank Accession No. NC_001631). Twenty five nanograms of each chloroplast probe and 100 ng of genomic DNA were labeled with ^32^P-dCTP using a random primer labeling technique [56], [57]. Labeling was performed at 37°C for 1–3 h and the unincorporated nucleotides were removed using the QiaQuick Nucleotide Removal kit.

Hybridization of probes to macroarrays, membrane washing, and visualization of positive hybridization sites were performed as described in Table S1.

### Southern blot

To further verify that the BAC library we constructed contained pine genomic DNA inserts, one of the gels used for insert size determination was transferred to a nylon membrane and probed with labeled pine genomic DNA as described previously [58].

### Sequencing of LP BAC Clone PT_7Ba_00066 Well J18

Approximately 1 µg of LP BAC Clone PT_7Ba_0006 J18 was prepared for sequencing on an Illumina GAII using custom barcoding adapters that enable multiplex template sequencing [59]. This specific LP BAC was barcoded with “AGCT” and represented ∼1/10 of the sample pool. Standard Illumina chemistry was used for cluster generation. Specifically, 5 pM of the multiplex library was subjected to 36-amplification cycles to give single-end sequencing reads [60]. The sequencing was performed at the Oregon State University Center for Gene Research and Biocomputing. A total of 6.62 million clusters passed purity filtering, with 387,855 clusters attributable to this LP BAC. Barcodes were removed *in silico* from the 5′ ends of microreads, and the remaining 32 bp microreads were assembled *de novo* with Velvet [61] using the following parameters: cov_cutoff  = 10; min_contig_lgth  = 75. Velvet produced 159 contigs ≥75 bp in length with an N50 of 2.97 kbp. Excluding vector sequence, these contigs had a cumulative length of 85.4 kb.

### BAC sequence analysis

The contigs from LP BAC clone PT_7Ba_00066 J18 were used as queries in BLAST (blastn) searches against plant sequences in the GenBank non-redundant (nr) and non-human, non-mouse EST (est_others) databases. The location of each hit with a bit score of 50 or greater was aligned with the contig sequence. Regions of the contig were manually classified based upon their top GenBank hits. To identify putative MITES, we used the contig sequences as queries for FINDMITE [35] with the TIR (terminal inverted repeat) length set at 11 and a tolerance of up to two bases mismatches per TIR.

### Characterization of the sequenced LEA gene

The LEA gene sequenced as part of this work (LBAC) was compared (blastn) with *P*. *taeda* ESTs in the non-human, non-mouse EST database (est_others). The EST sequences representing the top 250 hits were extracted and aligned with the LBAC using MUSCLE [62]. The NCBI BLASTX tool was used to compare the predicted amino acid sequence of the LBAC gene product with previously characterized proteins.

### Determination of copy number of repeat families

Repeat copy numbers were estimated from macroarrays as described in Peterson et al. [42] with modifications to account for false positives and clones containing chloroplast DNA (see Table S2 for calculations).

## Supporting Information

Figure S1 **Comparison of LBAC, the consensus sequences of the two major EST variants discovered through BLAST alignment (i.e., CSV1 and CSV2), and the *P*. *halepensis* LEA EST sequence (PHLE).** For each sequence, the start codon is highlighted in light blue, the stop codon in pink, and the intron (if any) in light orange. The exons in CSV1 are identical to those in the LBAC gene. 2.5% of the ESTs used to create CSV1 contained a putative intron with 100% sequence identity to the intron in LBAC (light orange highlight). CSV2 was derived from 11 sequences that showed significant and consistent differences from CSV1/LBAC. The region believed to represent an intron in CSV1/LBAC was present in all transcripts used in generating CSV2, and indeed it may be that all mature sequences produced from this locus/allele contain the “intronic” region (hence this region is not highlighted as an intron in CSV2). Compared to CSV1, CSV2 contains a deletion in the putative intron region (bases 225-236), which may account for improper splicing of the CSV2 transcript, and a 13 nt insertion immediately after the stop codon (bases 455-467). The 13 nt insertion is also observed in PHLE. Single nucleotide differences between a particular sequence and the LBAC sequence are highlighted in yellow.
(TIF)

Figure S2 **The LP LEA protein shows similarity to the LEA 3 family of proteins (pfam03242).**
(TIF)

Table S1 **Additional Methods.**
(DOCX)

Table S2 **Calculating the copy number and genome percentage of IFG-7 based on densitometric analysis of a macroarray.** Based on Peterson et al. [42] Supplementary Documents. Aqua shaded cells contain data generated in the current study. Violet shaded cells contain data from the literature.
(TIF)
