# Supplementary figures and images for: Adventures in the Enormous: A 1.8 Million Clone BAC Library for the 21.7 Gb Genome of Loblolly Pine
Source: PLoS One. 2011 Jan 21;6(1):e16214. doi: 10.1371/journal.pone.0016214 (PMC3025025; doi:10.1371/journal.pone.0016214)

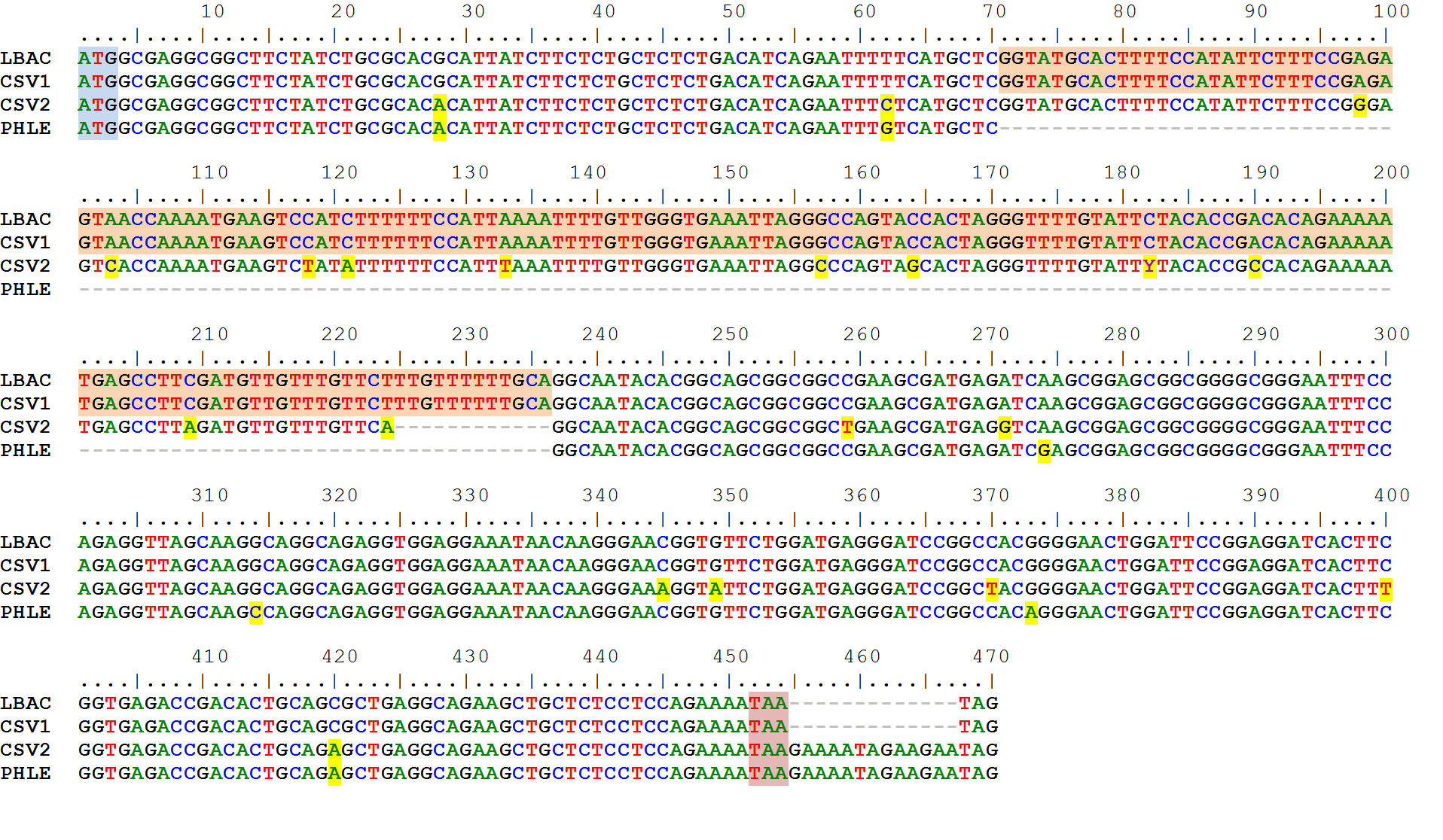

Supplement: Figure S1 — Comparison of LBAC, the consensus sequences of the two major EST variants discovered through BLAST alignment (i.e., CSV1 and CSV2), and the P. halepensis LEA EST sequence (PHLE). For each sequence, the start codon is highlighted in light blue, the stop codon in pink, and the intron (if any) in light orange. The exons in CSV1 are identical to those in the LBAC gene. 2.5% of the ESTs used to create CSV1 contained a putative intron with 100% sequence identity to the intron in LBAC (light orange highlight). CSV2 was derived from 11 sequences that showed significant and consistent differences from CSV1/LBAC. The region believed to represent an intron in CSV1/LBAC was present in all transcripts used in generating CSV2, and indeed it may be that all mature sequences produced from this locus/allele contain the “intronic” region (hence this region is not highlighted as an intron in CSV2). Compared to CSV1, CSV2 contains a deletion in the putative intron region (bases 225-236), which may account for improper splicing of the CSV2 transcript, and a 13 nt insertion immediately after the stop codon (bases 455-467). The 13 nt insertion is also observed in PHLE. Single nucleotide differences between a particular sequence and the LBAC sequence are highlighted in yellow. (TIF) [file pone.0016214.s001.tif]

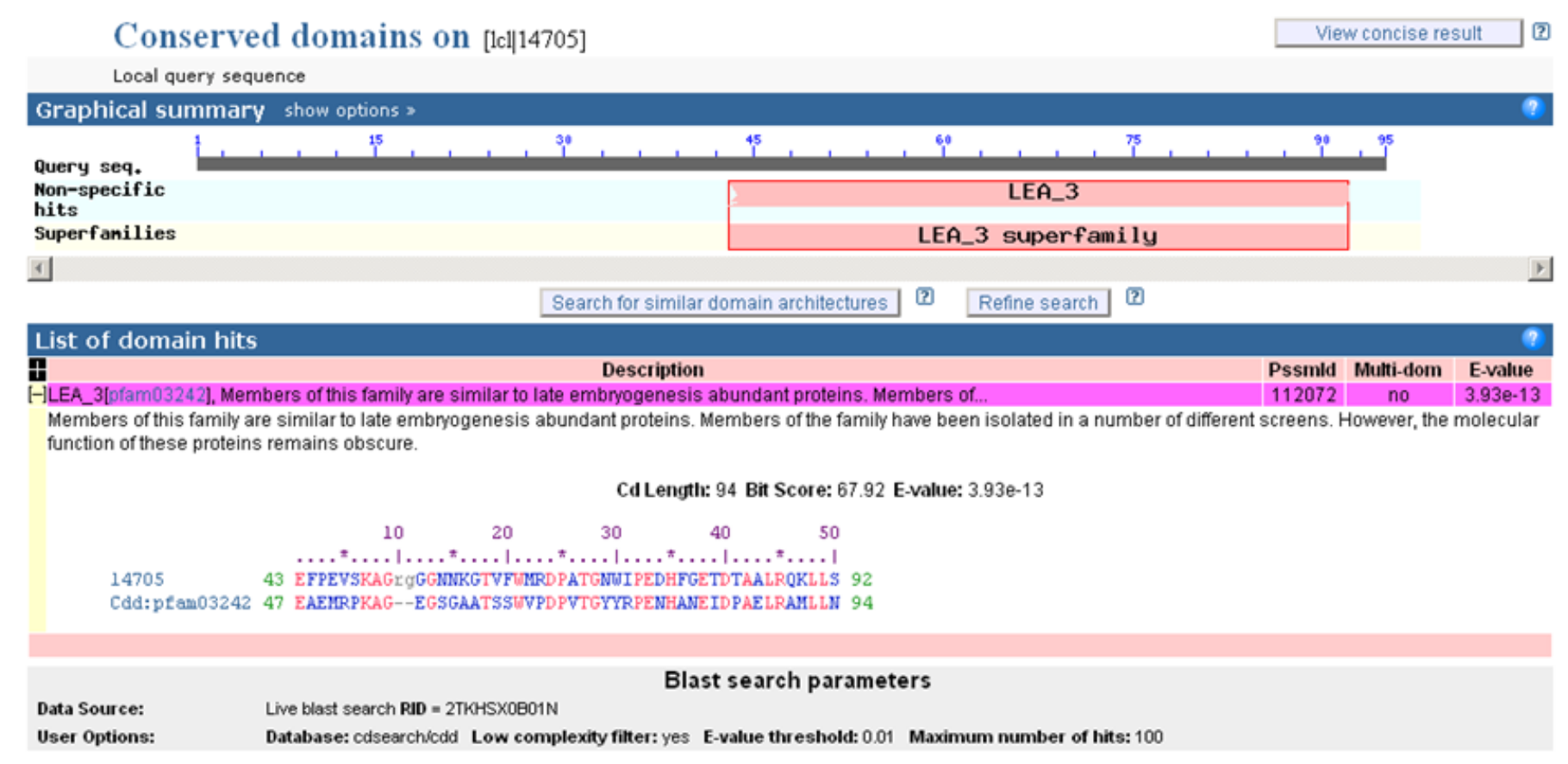

Supplement: Figure S2 — The LP LEA protein shows similarity to the LEA 3 family of proteins (pfam03242). (TIF) [file pone.0016214.s002.tif]

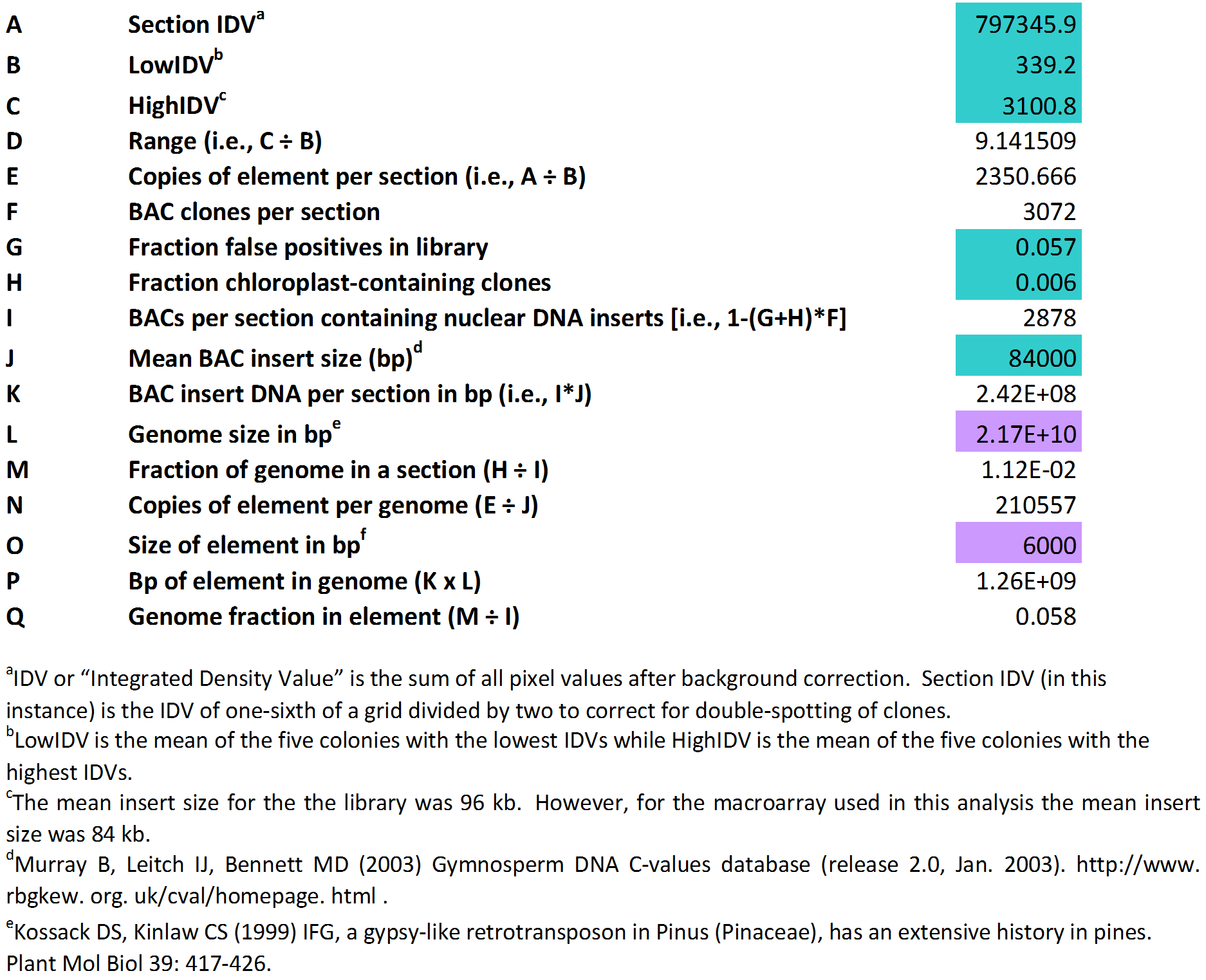

Supplement: Table S2 — Calculating the copy number and genome percentage of IFG-7 based on densitometric analysis of a macroarray. Based on Peterson et al. [42] Supplementary Documents. Aqua shaded cells contain data generated in the current study. Violet shaded cells contain data from the literature. (TIF) [file pone.0016214.s004.tif]
