# Supplementary material for: Adventures in the Enormous: A 1.8 Million Clone BAC Library for the 21.7 Gb Genome of Loblolly Pine
Source: PLoS One. 2011 Jan 21;6(1):e16214. doi: 10.1371/journal.pone.0016214 (PMC3025025; doi:10.1371/journal.pone.0016214)
Supplement: Table S1 — Additional Methods. (DOCX) [file pone.0016214.s003.docx]

| **Overgo design and preparation** | To design the overgos, we used the EST sequence of the selected gene to design an overgo probe. In short, a 36 bp, 50% GC, non-repetitive portion of the EST was selected using a custom Perl script developed at MGEL. This target sequence was split into two 22 bp primers representing the 5’ ends of the target. The primers were designed to possess 8-bp of sequence complementarity with 50% GC content at their 3’ ends. The primers were added together in equimolar concentrations (10 pmol each) in a total aqueous volume of 5.5 μL, heated to 95^o^C for 5 min, and allowed to anneal at 37^o^C. The 3’ ends of the partial duplexes were filled in with labeled and non-labeled nucleotides to produce 36-bp, double-stranded, overgo probes. The end-fill reaction consisted of the 5.5 μL of annealed primers, 0.5 μL of 2 mg/mL aqueous BSA, 2 μL of oligo labeling buffer without dATP or dCTP (Ross et al 1999), 0.5 μL each of α[^32^P]-dATP and α[^32^P]-dCTP (MP Biochemicals, Santa Ana, CA), and 1 μL of Klenow fragment (New England Biolabs). The mixture was incubated at 37^o^C for 2 – 3 h and unincorporated nucleotides were removed using the QiaQuick Nucleotide Removal kit (Qiagen). |
| --- | --- |
| **Overgo primers** | Forward: 5’ – ACACTGCAGAGCTGAGGCAGAA- 3’  Reverse: 5’ – TTCTAGATAGGAGCTTCTGCCT- 3’ |
| **PCR-based verification of positive clones** | The PCR mix was composed of 100 ng of purified template (positive BAC) DNA, 250 µM of each dNTP, 0.5 µM of each primer, 5 µL of 10X *Taq* buffer (New England Biolabs; 100 mM Tris-HCl, 500 mM KCl, 15 mM MgCl_2_, pH 8.3), 0.5 µL of 5 units/µL *Taq* polymerase (New England Biolabs or Takara), and water to produce a final total volume of 50 µL. The reaction was denatured at 94^o^C for 5 min followed by 30 cycles consisting of denaturation for 30 s at 94^o^C, incubation for 30 s at 50^o^C, and extension for 30 sec at 72^o^C. A final 5 min incubation at 72^o^C permitted extension of incomplete products. |
| **PCR primers** | Forward: 5’ – TATGGCAGGACGGCTTCTAT- 3’  Reverse: 5’ – GGTTGGGTCTCTCATCCAGA- 3’ |
| **Macroarray hybridization** | The labeled probe was denatured at 95^o^C for 5 min and incubated on ice for 5 min before adding to a pre-heated tube of hybridization buffer (1% BSA, 1 mM EDTA pH 8.0, 7% v/v SDS, 0.25M sodium phosphate pH 7.2). 10 mL of buffer was allotted per filter. The pre-hybridization buffer was discarded and replaced with pre-heated buffer containing the labeled probe. Hybridization was carried out at 55^o^C for overgos and 65^o^C for longer probes. Hybridization was carried out for about 42 h.  To prepare for washing, hybridization buffer was decanted into a glass beaker and the filters rinsed with about 200 mL of room temperature washing buffer (1X SSC and 0.1% w/v SDS). To remove non-specific signals, the filters were washed twice for 30 min with washing buffer at the temperature of hybridization (washing temperature). When background signal continued to be detected after two washes, a third wash was performed. The filters and the nylon mesh sheets were transferred into a large plastic container with 1 L of washing buffer equilibrated at the washing temperature, then rinsed individually into a second plastic container containing the same buffer and at the same temperature. The filters were blotted onto filter paper to remove excess liquid, wrapped in Saran wrap to maintain dampness, and exposed to storage phosphor screens (GE Healthcare) for 1 to 3 days. The screens were scanned with a Storm 820 (GE Healthcare) instrument. The images were converted into viewable files using ImageJ (<http://rsb.info.nih.gov/ij/index.html>) and analyses were carried out manually and with the use of our *MacroArray* *Reader* program. The processed images and coordinates of the hybridized spots were stored and deconvoluted via a local web interface/database.  The bound probes were removed from the filters by gentle shaking at room temperature in stripping buffer (100 mM NaOH, 10 mM EDTA pH 8.0 and 0.1% w/v SDS) in increments of 10 min until no signal was detected. In general, 1 or 2 stripping steps were sufficient. The filters were then rinsed with double-distilled water and shaken twice in 5X SSC for 10 min. The filters were blotted with filter paper, enclosed in Ziploc bags and stored at 4^o^C. |
